# Geo-temporal study of clinical malaria in an endemic zone in southern Mali: The case of the Kolondieba health district from 2019 to 2021

**DOI:** 10.5281/zenodo.15676301

**Published:** 2025-05-16

**Authors:** Ibrahima Berthé, Mady Cissoko, Mamady Koné, Donatien Serge Mbaga, Alou Diaby, Abdramane Konaté, Ismaila Théra, Bayaya Haidara, Abdoulaye Ongoiba, Tahirou Togola, Modibo Diarra, Ousmane Boua Togola, Amagoron dit Mathias Dolo, Souleymane Diarra, Bourahima Koné, Yacouba Koné, Lassana Sissoko, Leon Paul Rabarijaona, Cheick Abou Coulibaly, Cheick Amadou Tidiane Traore, Issaka Sagara

**Affiliations:** 1 Disease Prevention and Control Department and General Directorate of Health and Public hygiene, Ministry of Health and Social Affairs, Bamako, Mali.; 2 Malaria Research and Training Center (MRTC), Department of Epidemiology of Parasitic Diseases, Bamako, Mali.; 3 National Malaria Control Program, Bamako, Mali.; 4 Ecole Supérieure des Sciences de la Santé Siantou, Institut Universitaire Siantou, Yaoundé, Cameroon.; 5 Population Development and Reproductive Health Research and training Institute, Cheikh Anta Diop University, Dakar, Senegal.; 6 Kolodiéba District Hospital, Kolodiéba, Mali.; 7 Institut National de Santé Publique, Mali.; 8 Unicef Mopti Field Office, Mali.; 9 Department of Public Health, Faculty of Medecine and Odonto-Stomatology, University of Sciences, Techniques and Technologies of Bamako, Mali.

## Abstract

**Background:**

Malaria remains a significant public health challenge in Mali, particularly in endemic areas such as the Kolondieba health district. This study aimed to analyse the geo-temporal dynamics of clinical malaria transmission, identifying high-risk periods, vulnerable age groups and associated environmental and health determinants.

**Materials and Methods:**

A historical cohort study was conducted from 2019 to 2021 across 21 health facilities in the Kolondieba district. Epidemiological, climatic, and demographic data were analysed using geospatial tools (QGIS) and statistical software (R). The non-parametric Wilcoxon and Kruskall-Wallis tests were used to compare two means and population malaria incidence distribution, respectively.

**Results:**

The incidence of malaria exhibited seasonality influenced by precipitation and humidity, while elevated temperatures were associated with a decrease in malaria incidence. Periods of high transmission potential (HTP) last for 20-25 weeks annually (weeks 23-48) and peak around weeks 30-31. Malaria accounted for 53.71% of consultation reasons, with pronounced vulnerability observed in children aged 0-4 yrs, especially during high transmission periods. Spatial stratification revealed two risk levels: 5 health areas at moderate risk (incidence 251-450 cases/1000 inhabitants) and 16 at high risk (>450 cases/1000 inhabitants). Health center attendance was a more determining risk factor.

**Conclusion:**

This study highlights the spatial and temporal heterogeneity of malaria transmission in southern Mali, emphasising the necessity to target interventions during weeks 23-48 (June through November), among children <5 yrs of age, in health areas with high health centre attendance. The integration of socio-economic factors in future studies could refine control strategies.

## Introduction

Malaria, a parasitic disease transmitted by *Anopheles* mosquitoes, remains a major global health challenge, particularly in tropical and subtropical regions [1,2]. Malaria is caused by the *Plasmodium* parasite, of which the *P. falciparum* is the most virulent and prevalent in Africa; malaria continues to place a heavy burden on health systems, especially in resource-limited countries [3–5]. In 2024, the World Health Organization (WHO) recorded nearly 263 million cases of malaria worldwide, with 94 per cent concentrated in sub-Saharan Africa. Children <5 yrs of age and pregnant women remain the most vulnerable groups. In Mali, one of world’s 11 most affected countries, malaria constitutes the leading cause of consultation in health facilities, according to the 2022 statistical yearbook [6,7].

Indeed, malaria transmission is influenced by a complex combination of environmental, climatic and behavioural factors. Climatic conditions such as abundant rainfall and high temperatures create environments conducive to mosquito breeding and accelerate parasite development within the vector [8,9]. Furthermore, environmental factors, including land use patterns, vegetation and proximity to waterbodies, may also have an impact on mosquito populations and vectorial capacity [10,11]. Finally, human factors, such as socio-economic status, access to healthcare and the use of preventive measures such as insecticide-treated bednets, signifcantly influence the risk of contracting the disease [12].

Despite the efforts deployed to control and eliminate malaria, the persistence of the disease underscores the necessity for more targeted and tailored approaches. Within its global strategic plan aimed at reducing malaria morbidity and mortality by 90%, the WHO encourages endemic countries to conduct malaria risk stratification. This strategy relies upon an update of vulnerable groups, which may evolve with the intensification of interventions, and upon the identification of optimal periods to implement adapted actions [13]. Such an approach enables a better structuring of malaria control, by targeting the areas and populations most at risk.

Current interventions, such as vector control, early diagnosis followed by prompt treatment with artemisinin-based combination therapies (ACT), and seasonal malaria chemo-prevention (SMC), have demonstrated their efficacy in reducing disease severity, preventing transmission, and decreasing mortality [14–17]. Recent vaccination against malaria with the R21 or RTS,S vaccines opens up new perspectives in malaria prevention. However, to optimise these interventions, a thorough understanding of the spatial and temporal dynamics of transmission is essential.

Recent advances in geospatial modelling, combining Geographic Information Systems (GIS) and remote sensing, have revolutionised the study of vector-borne diseases such as malaria [18]. GIS enables the overlay of information layers (malaria case distribution, mosquito density, healthcare access) to generate high-resolution predictive maps [19]. In Senegal, a machine learning model using rainfall and local data has helped to predict malaria epidemics, enabling targeted and more effective interventions [20].

It is within this context that this study focused on the geospatial analysis of clinical malaria in the Kolondieba health district of Mali, a region characterised by high malaria transmission rates, where precise data on risk factors will contribute to reducing the malaria burden.

The objective of this study was to perform a geo-temporal analysis of clinical malaria by identifying the most vulnerable age groups, high-risk malaria areas, as well as the environmental and health determinants associated with disease transmission.

### Materials and Methods

**Figure 1:**
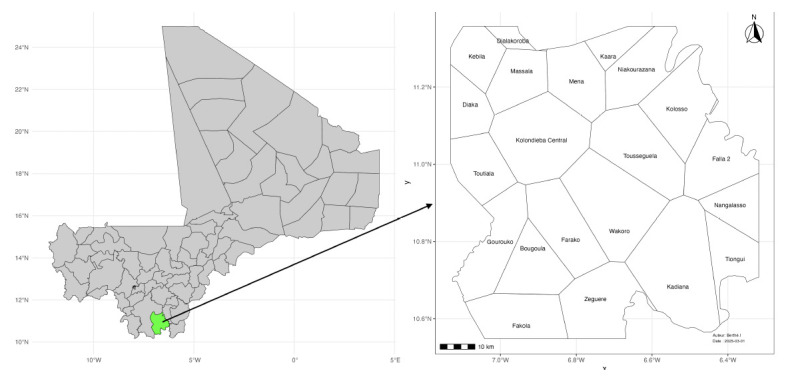
Kolondieba district in Mali (green, left image) and the district’s health areas (right image).

A historical malaria routine data study was conducted over a period of three (3) years, (1 January 2019 to 31 December 2021). It was carried out in the Kolondieba health district, a malaria-endemic area located in the Sikasso region of southern Mali. This district covers an area of 9,200 km^2^and encompasses 21 health areas, with a total population of 296,394 inhabitants in 2021 [21] (Figure 1).

This location was selected because it is one of the districts with the highest malaria incidence in Mali, making it a relevant location to assess the effectiveness of interventions. Moreover, its status as a highly endemic area justifies its selection in a clinical trial, sponsored by Notre Dame University (USA) aimed at reducing disease transmission using spatial repellents against mosquitoes [22].

### Study population and inclusion criteria

The target population of the study encompassed all residents within the Kolondieba Health district who presented for care consultation at community health facilities and the reference district hospital of Kolondieba. All cases of malaria, confirmed by rapid diagnostic test (RDT) or malaria microscopy and recorded in the consultation registers of health facilities within the health district of Kolondieba were included.

### Data collection

Data were categorised into three principal groups: epidemiological, climatic and demographic.

Epidemiological data were collected from the consultation registers of 21 health areas within the health district, covering the study period. Information gathered included age, sex, occupation, address, clinical signs, biological malaria test results, administered treatment, and consultation frequency. A pre-defined questionnaire was parameterised within the KoboCollect tool to facilitate data entry by investigators.

In Mali, several mechanisms have been implemented to strengthen the quality of routine malaria data such as regular supervision at community health facilities (CSCom) to assess the correct use of rapid diagnostic tests (RDTs) and microscopy, the concordance between diagnosis and treatment, and the completeness of registration in the registry. To ensure data consistency, biannual analysis and validation sessions are organised at the regional and national levels, with verification of primary data as needed. Validated data are then integrated into DHIS2, the national health information system platform. In this study, emphasis was placed on the exploitation of individual data from consultation registers to minimise errors.

To minimise human errors, two experienced investigators were trained to use KoboCollect, an application that enforces constraints (required fields, value ranges) to reduce incorrect entries. Climatic data were extracted at the health area level, considering their geographical delineation obtained from the OpenStreetMap (OSM) tool. Climatic variables collected were extracted from the Nasa Giovanni website using satellite databases (MERRA-2, MODIS Terra, GLDAS) at the required spatial resolutions (0.1° to 0.625°). Internal validation of these data is documented in the scientific literature (Table 1).

**Table 1:** Variables and sources.

Variables	Scale	Units	Sources	Spatial resolution
Confirmed cases of malaria	Daily	-	Consultation register	-
Late cumulative precipitation	Daily	mm/day	GPM	0.1°
Average relative humidity	Daily	kg/m^2^	GLDAS Model	0.25°
Maximum temperature	Daily	°C	MERRA-2	0.5 x 0.625°
Average temperature	Daily	°C	MERRA-2	0.5 x 0.625°
Minimum temperature	Daily	°C	MERRA-2	0.5 x 0.625°
Vegetation	Annual	NDVI	MODIS Terra	0.05

Demographic data were extracted from the District Health Information Software 2 (Dhis2), the official platform of the Ministry of Health, which is regularly updated. These data included population distribution by health area for the study years.

Data obtained at the district and daily scales were aggregated to a weekly scale. For malaria cases and precipitation, the weekly sum was calculated. For surface relative humidity, as well as for maximum, mean, and minimum temperatures, the weekly mean was calculated. The weekly incidence of malaria was determined by dividing the sum of new weekly cases by the population per 1,000 persons. Thus, the weekly time series from 2019 to 2021 at the district scale of malaria incidence, late cumulative precipitation, surface relative humidity, and maximum, minimum, and mean temperatures were analysed to determine trends and seasonality. Change Point Analysis (CPA) of weekly series of malaria incidence was then performed using the Pruned Exact Linear Time (PELT) algorithm implemented in R to detect statistically significant shifts in means, thereby delineating distinct malaria transmission periods [23,24].

To identify at-risk age groups, the total number of malaria cases was calculated by age group, by year, and by transmission period (high and low). Malaria incidence was then determined for each age group, expressed as the number of cases per 1,000 inhabitants. The age groups of 0-4, 5-9, 10-14, 15-24, and over 24 years from the malaria surveillance guide in Mali were used. Thus, for each age group, the weekly mean of incidence per 1,000 inhabitants was calculated by study year, and then specifically over all periods of high and low transmission. Subsequently, the estimated mean incidences by age group were compared between the different study years, between the periods of high and low transmission, as well as within the same year, using multivariate analyses. For comparing two population malaria incidence distributions, the non-parametric Wilcoxon test was used, whilst the Kruskall-Wallis test was applied for comparisons involving several means of populations malaria incidence distribution.

The spatial analysis focused on the data from the last study year (2021). To determine the spatial variability of malaria transmission, the following variables were calculated for each health area: 1. Malaria proportion: the ratio of confirmed malaria cases to the total number of consultations; 2. Gross annual incidence: the number of confirmed cases divided by the total population, expressed per 1,000 inhabitants; 3. Attendance rate: the total number of new consultations divided by the population of the health area; 4. Adjusted cases: the number of confirmed cases divided by the attendance rate, to correct for disparities in access to health facilities; 5. Adjusted incidence: adjusted cases divided by the exposed population, expressed per 1,000 inhabitants;

Malaria risk stratification was performed according to the WHO standardised malaria elimination classes, applied to crude incidence and then adjusted to minimise bias related to disparities in access to care. Four categories were defined: 1. Very low risk: incidence < 100 cases per 1,000 population; 2. Low risk: incidence between 100 and 250 cases per 1,000 population; 3. Moderate risk: incidence between 251 and 450 cases per 1,000 population; 4. High risk: incidence > 450 cases per 1,000 population.

To produce a risk area classification map, the geographical coordinates of the health areas were projected onto the district layer in Quantum Geographic Information System (QGIS), enabling the random creation of health area-scale layers using the Voronoi diagram. Subsequently, the combination of the district layer and the data from the classification was used in R.

A significance threshold of p< 0.05 was retained for all tests for both temporal and spatial analysis. Thus, any p-value below this threshold was considered statistically significant. The statistical tests performed were considered significant if the p-value is less than 0.05.

R software version 4.1.1 was utilised for the analysis of the data. QGIS software version 3.10.3 was used to derive the health area layers from the Voronoi polygon. The following packages from the R software were used to conduct the analysis: "timeSeries", "CPA", "prop.test() and conf.level", "ggplot2", "table()", "ctree", "gam", "geom_sf".

### Ethical considerations

The protocol for this study was approved by the Ethics Committee of the Faculties of Medicine and Odontostomatology, and Pharmacy of the University of Sciences, Techniques, and Technology of Bamako (USTTB) under number 2020_252_CE/ FMOS/FAPH. The health and administrative authorities of Kolondieba were informed about the contents of the protocol and gave their permission for the collection and use of data from the health areas. To ensure patient confidentiality, no personally identifiable information was collected during the study.

## Results

A total of 117,942 confirmed malaria cases from the consultation registers for the years 2019-2021 from 21 health areas in the Kolondieba district were collected and analysed.

### Time series analysis of malaria incidence and climate data

**Figure 2:**
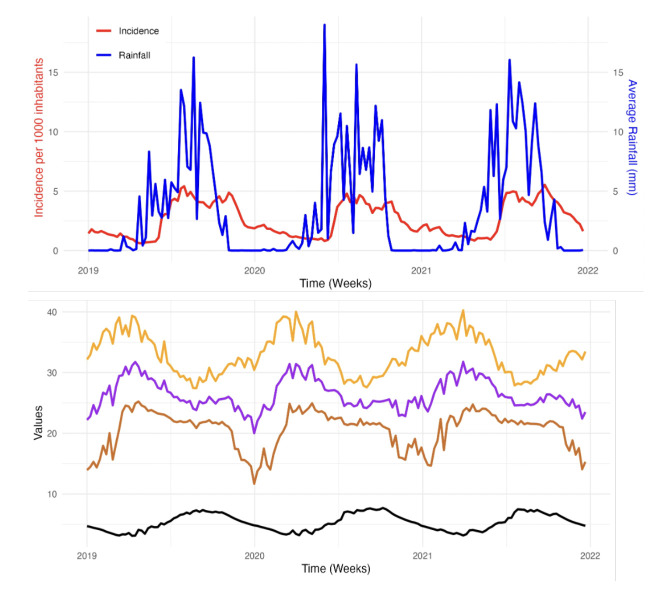
Top: Weekly time series of malaria incidence (red line) and rainfall (blue). Bottom: Average maximum (orange), mean (purple), and minimum (brown) temperatures (oC) and relative humidity (kg/m^2^) in Kolondieba Health District for the period 2019-2021.

Figure 2 shows the weekly malaria incidence per 1000 inhabitants alongside the weekly precipitation (in millimeters) over the period 2019-2021. The weekly malaria incidence remained relatively stable during these three years, ranging from 1 to 5 cases per 1000 inhabitants per week. The temporal distribution of malaria cases is characterised by two transmission periods, one of low intensity and another of high intensity. The high transmission period generally begins in the 23rd week and reaches a first peak in the 31st week of each year, ending around the 48th week. Concurrently, the precipitation curve also exhibits seasonality, with a systematic onset of rains around the 18th week and a cessation around the 45th week. The data reveal a seasonality and a similar annual evolution for these meteorological parameters. An increase in temperatures is observed from weeks 5, 7, and 1 respectively for the years 2019, 2020, and 2021, followed by a systematic decrease around the 26th week. The highest temperatures are generally recorded around the 15th week, frequently exceeding 40°C for the maxima. Conversely, the relative humidity increases from weeks 25, 27, and 26 respectively in 2019, 2020, and 2021, and then decreases around the 48th week for the three study years. It varies between 3 and 8 kg/m^2^per week, with extreme peaks observed respectively in weeks 37, 36, and 34 in 2019, 2020, and 2021 (Figure 2).

### Malaria transmission periods in the Kolondieba District (2019-2021)

The time series analysis of the incidence identified periods of high transmission lasting 20 to 25 weeks per year, encompassing two annual transmission peaks. The first peak consistently occurred around weeks 30 and 31. Over the three years studied, the high transmission period varied: In 2019, it lasted from week 23 to week 48; in 2020, it was shorter, spanning from week 26 to week 46; in 2021, it decreased slightly further, starting in week 26 and ending in week 45.

**Figure 3:**
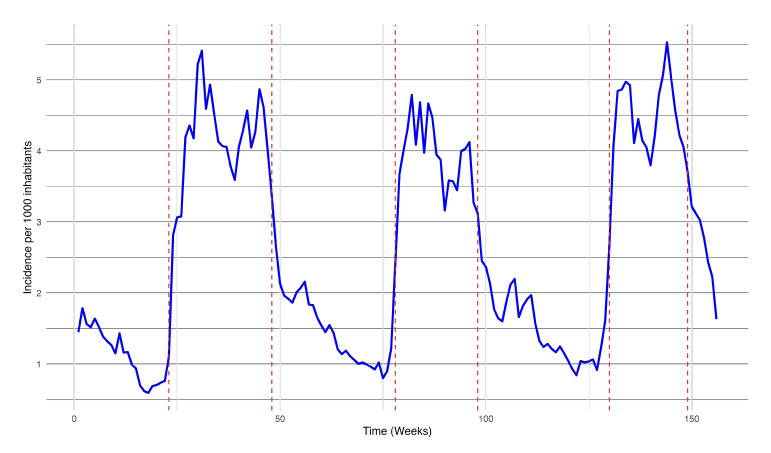
Determination of malaria transmission periods using Change Point Analysis of the mean incidence from 2019 to 2021 in the Kolondieba Health District.

These periods, visually marked by the red dotted lines in Figure 3, indicate the elevated transmission periods and the corresponding weekly incidence fluctuations (per 1,000 inhabitants).

### Malaria incidence stratified by age, year, and transmission period

**Figure 4:**
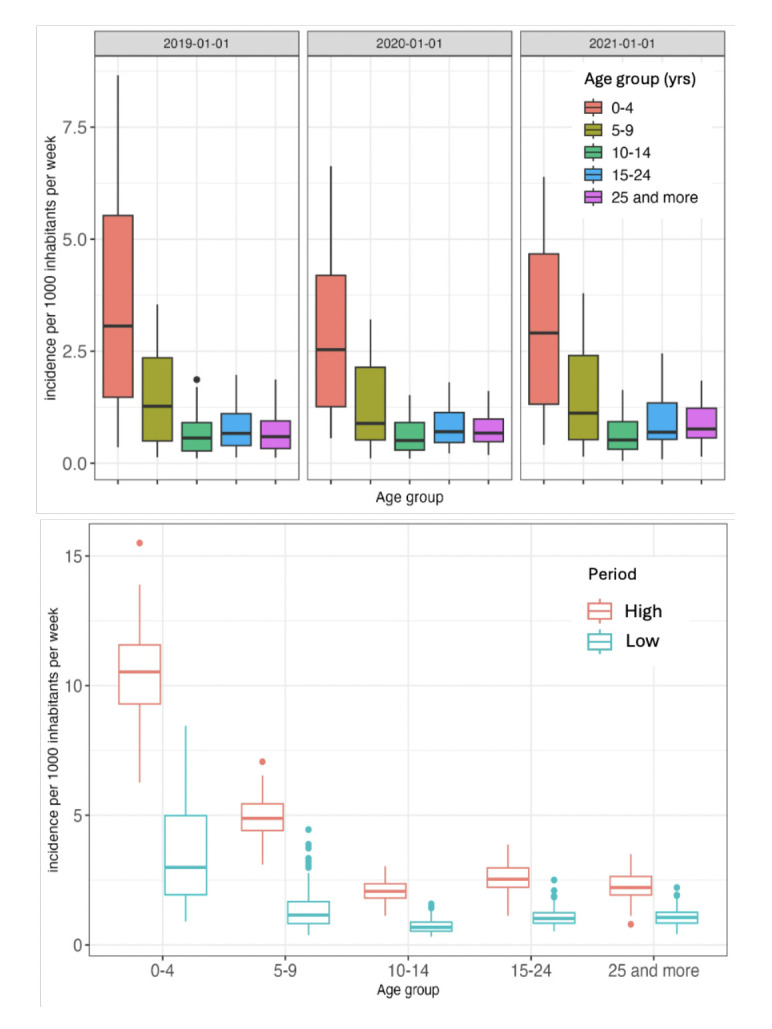
Malaria incidence by age group and year (top) and period of transmission (bottom) in the Kolondieba health district.

The top section of figure 4 represents the distribution, stratified by age group and by year, of malaria incidence per 1,000 inhabitants within the Kolondieba health district. In 2019, the overall incidence of malaria reached its highest level among the three years studied. The age groups of 0 to 4 years and from 5 to 9 years exhibited the highest incidences and the greatest variability, as evidenced by their elevated median and interquartile range (IQR) compared to the other age groups..

The bottom section of Figure 4 illustrates the cumulative incidence of malaria (per 1,000 inhabitants per week) over three study years, stratified by age group and by transmission period within the health district. A distinct variation in incidence is observed between the high and low transmission periods. A negative correlation between age and malaria incidence was observed. The 0-4 yrs age group presents the highest median incidence, reaching approximately 10 cases per 1,000 inhabitants per week during the high transmission periods, as well as the largest IQR amplitude across both transmission periods. Incidences progressively decrease in older age groups, with proportionally lower medians and IQRs for both periods.

It is also noteworthy that high outlier values are present in the 0-4 yrs age group, exceeding 15 cases per 1,000 inhabitants per week, whereas the other age groups exhibit less pronounced outliers.

### Comparison of incidence by age group and by year

For the youngest age group (0-4 yrs), the incidence was highest in 2019 (7.01) and lowest in 2020 (5.74). However, the p-value of 0.277 indicates the absence of a statistically significant difference between the three years. Similar trends were observed for the age groups 5-9 yrs and 10-14 ys) where incidence rates remained relatively stable, with high p-values (0.978 and 0.788 respectively). The 15-24 yrs age group showed minor fluctuations, with a slightly higher incidence in 2021 (1.88) compared to 2019 (1.57) and 2020 (1.61), however, the difference was not statistically significant (p = 0.067).

Conversely, the oldest age group (25+ yrs) exhibited a statistically significant difference (p = 0.004) in incidence over the years, increasing from 1.37 in 2019 to 1.78 in 2021. Furthermore, Table 2 reveals p-values <0.0001 for each year, indicating statistically significant differences in incidences between age groups within each year.

**Table 2:** Variability of weekly crude malaria incidence per 1,000 inhabitants according to age (intra and inter-annual).

		Years		
Age group (yrs)	2019	2020	2021	P value
0-4	7.01	5.74	6.19	0.277
5-9	2.92	2.63	2.93	0.978
10-14	1.28	1.25	1.32	0.788
15-24	1.57	1.61	1.88	0.067
25+	1.37	1.51	1.78	0.004
P value	<0.0001	<0.0001	<0.0001	

Between 2019 and 2021, the mean weekly incidence of malaria per 1,000 inhabitants within the Kolondieba district increased significantly (p< 0.0001) during the period of high transmission, across all age groups. This increase is particularly marked within the 0-4 yrs age group, where the incidence rose from 3.5 to 10.3 (Table 3).

**Table 3:** Variability of crude malaria incidence per 1,000 inhabitants between transmission periods by age group in the Kolondieba district (2019-2021).

Age group (yrs)	Period (Low)	Period (High)	P-value
0-4	3.5	10.3	<0.0001
5-9	1.4	4.9	<0.0001
10-14	0.7	2.6	<0.0001
15-24	1.1	2.6	<0.0001
25+	1.1	2.3	<0.0001

### Malaria among care seekers in health areas

At the health district level, the proportion of malaria in health care seekers was 53.71 % (95% confdence interval: 53.50 – 53.91), exhibiting local variability. The Diaka health area reported the highest rate with 91.38 % (95% confidence interval: 90.3 – 92.35), whereas the Kebila health area reported the lowest rate with 41.20 % (95% confidence interval: 40.37 – 42.04) (Table 4).

**Table 4:** Proportion of malaria cases out of all consultations by health area from 2019-2021 (Malaria Cases/ Total Number of Consultations) in Kolondieba health district

Health area	Total consultations	Malaria cases	Malaria burden (%)	Confidence interval (%)
Bougoula	12294	7505	61.04	60.18 - 61.91
Diaka	2993	2735	91.37	90.30 - 92.35
Dialakoroba	4198	2478	59.02	57.52 - 60.52
Fakola	13197	6663	50.48	49.63 - 51.35
Falaii	2366	1442	60.94	58.94 - 62.91
Farako	10394	7093	68.24	67.33 - 69.13
Gourouko	4075	1749	42.92	41.40 - 44.46
Kaara	4789	2084	43.51	42.11 - 44.94
Kadiana	48490	27650	57.02	56.58 - 57.46
Kebila	13458	5545	41.20	40.37 - 42.04
Kolondieba central	25085	10828	43.16	42.55 - 43.78
Kolosso	13445	8991	66.87	66.07 - 67.67
Massala	2292	1090	47.55	45.50 - 49.63
Mena	5877	4209	71.61	70.44 - 72.76
Nangalasso	5247	3058	58.28	56.93 - 59.62
Niakourazana	4261	2132	50.03	48.52 - 51.55
Tiongui	8193	3888	47.45	46.37 - 48.54
Tousseguela	13816	5996	43.39	42.57 - 44.23
Toutiala	12171	6486	53.29	52.40 - 54.18
Wakoro	6648	3268	49.15	47.95 - 50.37
Zegiiere	6467	3053	47.20	45.99 - 48.43
District	219586	117942	53.71	53.50 - 53.91

### Risk stratification

Risk malaria stratification applied to the crude incidence classifies the majority of health areas as very low/low risk (17/21: 81%). After adjusting the incidence on the health attendance rate, no health area is classified in the very low/low transmission risk category, all are classified in the category of health areas with moderate/high transmission risk (21/21: 100%) (Table 5).

**Table 5:** Classification of health areas according to WHO standardised thresholds based on incidence.

Stratification	WHO Class (incidence/ 1000/yr)	Number of health areas	WHO Class (adjusted incidence/ 1000/yr)	Number of health areas
Very low	<100	6	<100	0
Low	100-250	11	100-250	0
Moderate	251-450	3	251-450	6
High	>450	1	>450	15

**Figure 5:**
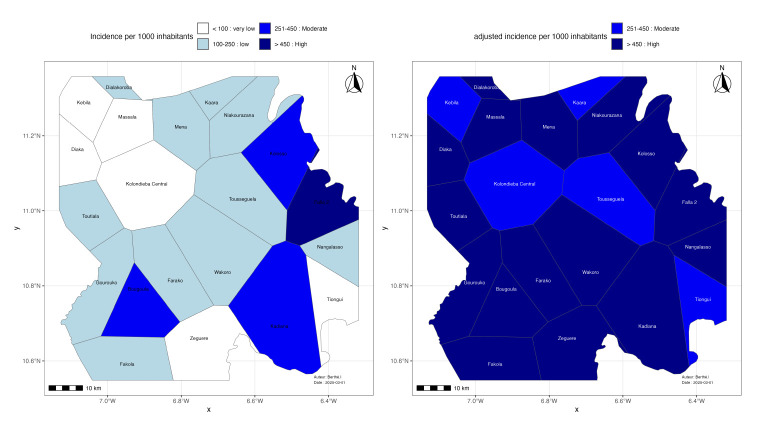
Malaria incidence (left) according to WHO classification and adjusted incidence (right) in the 21 health areas of Kolondieba health district.

The crude malaria incidence map of the spatial distribution of risk shows heterogeneity at four levels (Figure 5). Four health areas show dark shades, indicating moderate to high risk (crude incidence >250 cases/1,000 inhabitants/year) concentrated in the western part of the district. The adjusted malaria incidence map shows partial homogeneity of transmission risk at two levels with almost all health areas shifting towards dark shades, indicating moderate to high risk (adjusted malaria incidence >250 cases/1,000 inhabitants/ year) across the entire district.

## Discussion

The time series analysis within the Kolondieba district from 2019 to 2021 revealed a relatively stable annual incidence of malaria over the three years, exhibiting seasonality influenced by meteorological factors, albeit with a lag of several weeks.

Precipitation correlated with an increase in malaria incidence, whereas elevated temperatures correlated with a decrease in malaria incidence, likely due to the inhibition of vector development. Conversely, high humidity, often associated with increased precipitation, fostered mosquito proliferation and parasite development, leading to a rise in malaria cases.

The warmest period, around week 15, coincided with the lowest humidity levels, whereas the increase in humidity during the latter part of the year was accompanied by a decline in temperatures. These observations are consistent with the findings of Ogunsakin et al. [25] in Nigeria, who emphasised the involvement of meteorological factors, such as precipitation, temperature, and humidity, in the transmission dynamics of malaria. Furthermore, similar studies conducted by Katile *et al.* [26] in Kati, Sissoko *et al.* [27] in Bamako, Rouamba *et al.* [28] in Burkina Faso, and Ouedraogo *et al.* [29] in Ouagadougou, have also demonstrated a temporal relationship with delays ranging from 2 to 9 weeks between precipitation, humidity, temperature, and malaria incidence. These temporal variations underscore the importance of considering local specificities for accurately modelling and predicting malaria transmission dynamics. Such an approach would enable the adaptation of control and prevention strategies based on local contexts, thereby optimising their effectiveness.

The analysis of change points in the weekly incidence of malaria within the Kolondieba health district (2019-2021) highlighted statistically signifcant seasonal trends, characterised by periods of high transmission (HTP) dynamic depending on the year (in 2019, it lasted from week 23 to week 48, in 2020, it went from week 26 to week 46 and in 2021, it lasted from week 26 to week 45).

This analysis revealed a lag between HTP and the dates of SMC campaigns in the Kolondieba district. Although SMC is rigorously conducted between week 26 and week 40, HTP extends to weeks 45-48 depending on the year (2019-2021). This lag leaves a window of vulnerability where transmission persists after interventions have stopped, a phenomenon also observed by Cairns *et al.* [30] in Burkina Faso and Mali that recommends a fifth round of monthly SMC to adequately cover the whole transmission season in the study areas and in settings with similar epidemiology.

The relative decrease in the incidence curve in 2020 could be partly related to the mass distribution campaign of long-lasting insecticidal nets (LLINs) conducted that year. This protective effect is consistent with the findings of Fernandez Montoya *et al.* [31] who demonstrated in Mozambique the reduction of exposure to mosquitoes with the use of LLINs [31].

These results align with the expected precipitationinduced malaria transmission dynamics in the Sudano-Sahelian zone, confirming the major influence of local climatic conditions on the epidemiology of the disease. The quantitative data generated by this study provide precise information on the period and duration of maximum malaria risk, which would be essential for optimising public health interventions. For instance, the distribution of LLINs and the implementation of SMC could be planned more strategically to coincide with the onset and peak of HTP, thereby maximising their impact.

Regarding the periods of high transmission, these results differ from those reported by Cissoko *et al.* [32] in Diré, Mali in 2020, where the period of high transmission begins in August. This divergence suggests the existence of local variations in malaria transmission dynamics, likely influenced by environmental, climatic, or socio-economic factors specific to each region.

The analysis of the weekly malaria incidence rate per 1000 inhabitants, stratified by age, revealed an inverse relationship between age and malaria risk, with the latter progressively decreasing in older age groups. Children aged 0-4 yrs exhibit the highest median incidence and the widest interquartile range (IQR) over the three study years, reflecting risk heterogeneity and vulnerability within this group. These observations are consistent with those of Mpimbaza *et al.* [33] in Uganda. Conversely, older age groups display progressively lower median incidences and IQRs, indicating a more stable and less elevated malaria burden.

Seasonal variations in incidence show differences between high and low transmission periods. These fluctuations are particularly pronounced in children aged 0-4 yrs, suggesting increased vulnerability and variability during high transmission periods. These results are similar to those of Griffin *et al.* [34] and the systematic review by Carneiro *et al.* [35], all in sub-Saharan Africa. Notably, the cumulative three-year median incidence during the low transmission period for the 0-4 yrs age group exceeds that of the high transmission periods for older age groups (10-14 yrs, 15-24 yrs, and 25+ yrs). This indicates that young children play a central role in maintaining malaria transmission in the district.

These results are consistent with those of Cao *et al.* [36] in high-risk regions worldwide, who observe significant variations in malaria incidence by age and season. These similarities reinforce the idea that young children, particularly those <5 yrs, constitute a key population for malaria control interventions in endemic areas. This result could be explained by an unintended consequence of free malaria treatment for children below five years of age. Indeed, this incentive measure may encourage higher healthcare utilisation among this age group, leading to more systematic case detection. In contrast, other age groups, less targeted by such policies, might experience underreporting of the actual disease burden. Thus, the observed disparity would reflect not a true epidemiological difference but rather a diagnostic bias linked to healthcare accessibility.

The study by Kazanga *et al.* [37] in Kedougou, Senegal, confirms this hypothesis by demonstrating that children under SMC had a lower overall incidence compared to older children and young adults, highlighting the effectiveness of this intervention in this vulnerable group.

The comparison of the average weekly incidence per 1000 inhabitants between the age groups 0-4 yrs, 5-9 yrs, 10-14 yrs, and 15-24 yrs over the 2019-2021 period reveals no statistically significant difference between the three years. This stability suggests that incidence rates by age group remained relatively constant over this period. However, a statistically significant difference (p = 0.004) was observed for the oldest age group (25+ yrs), indicating an increase in incidence over the years. This trend could be explained by an increase in the vulnerability of this age group or by an epidemiological change.

Furthermore, the comparison of incidence rates between age groups within each year revealed statistically significant differences (p < 0.0001) for all years studied. This confirms that age groups are affected differently, highlighting the importance of considering age-related disparities in epidemiological analysis.

From 2019 to 2021, the average weekly incidence per 1000 inhabitants increased significantly during high transmission periods in all age groups. However, this increase was particularly marked in the 0-4 yrs age group, demonstrating increased vulnerability in young children compared to other age groups. These results underscore that, although all age groups are exposed to increased risk during high transmission periods, children aged 0-4 yrs constitute a more vulnerable population [36].

The malaria-related morbidity burden presents at levels exceeding 40% across all health areas, with disparities indicative of a non-uniform infection risk. This rate surpasses the national average, which stood at 29.00% and 39.26% in 2020 and 2022 respectively, according to the statistical yearbooks of the Mali local health information system [6,38]. These local disparities likely arise from variations in environmental exposures, socio-economic conditions, access to healthcare, demographics, and other risk factors.

Our stratification analysis according to WHO thresholds revealed a marked divergence between gross and adjusted incidences, reflecting two different epidemiological realities: a) Gross incidence: Initial data show heterogeneity across four strata, with 81% of areas classified as very low/low risk (<250 cases/1,000 inhabitants/year). This result suggests an illusion of control, similar to the observations of Alonso *et al.* [39] in rural West Africa, where under detection linked to limited access to care artificially minimises reported morbidity; b) adjusted incidence: After correction for attendance rates, partial homogeneity emerged, with all health areas reclassified as moderate/high risk (>250 cases/1,000 inhabitants/year). This trend corroborates with the estimates of Bhatt *et al.* [40], according to which 60% of sub-Saharan cases are under-diagnosed. The divergence between these measures underlines the crucial importance of adjusting incidence by the rate of attendance at health facilities, in accordance with the WHO guidelines (2020).

Although land use is similar across all health areas, notably through the practice of agriculture, disparities were observed. These differences could be explained by the presence of gold panning sites, which attract seasonal workers, influencing local transmission. This highlights the role of population mobility, which could bias local incidence.

A notable observation revealed that health areas exhibiting high health consultation service attendance rates are associated with an increased risk of malarial infection, suggesting improved diagnosed morbidity.

## Limitations

The non-availability of all consultation registers from certain CSComs precluded the collection of all data from 7 of the 21 health areas, corresponding to 7.5% of weekly data. Imputation was performed to account for this gap. The annual data for 2021 from the Kaara health area, included in the stratification, were extracted from Dhis2 instead of health registry data. Clinical malaria cases from certain private structures were not collected due to the lack of correct archiving of registers.

## Conclusions

The results of our study demonstrated spatio-temporal variability in malaria transmission, characterised by a period of elevated transmission (weeks 23-48), and two classes, i.e., 5 health areas classified as moderate and 16 as high. The rate of health facilities attendance was identified as a more influential determinant of the risk of malaria infection. Children aged 0-4 yrs were found to be the most vulnerable age group for clinical malaria throughout the year, with a notable exacerbation during periods of high transmission.

These observations underscore the necessity to target prevention and control interventions towards the periods, areas, and populations at greatest risk. The upcoming seasonal vaccination with R21 also offers a new opportunity. A complementary study integrating socio-economic factors could deepen the understanding of transmission determinants and refine the risk classification. This approach would enable the optimisation of public health strategies to reduce the burden of malaria within this endemic region.
